# Reproducing on Time When Temperature Varies: Shifts in the Timing of Courtship by Fiddler Crabs

**DOI:** 10.1371/journal.pone.0097593

**Published:** 2014-05-15

**Authors:** Kecia A. Kerr, John H. Christy, Zoé Joly-Lopez, Javier Luque, Rachel Collin, Frédéric Guichard

**Affiliations:** 1 Department of Biology, McGill University, Montréal, Québec, Canada; 2 Smithsonian Tropical Research Institute, Balboa, Ancon, Panama, Panama; 3 McGill-STRI Neotropical Environment Option (NEO), McGill University, Montréal, Québec, Canada; 4 University of Alberta, Department of Biological Sciences, Edmonton, Alberta, Canada; College of Charleston, United States of America

## Abstract

Many species reproduce when conditions are most favorable for the survival of young. Numerous intertidal fish and invertebrates release eggs or larvae during semilunar, large amplitude, nocturnal tides when these early life stages are best able to escape predation by fish that feed near the shore during the day. Remarkably, some species, including the fiddler crabs *Uca terpsichores* and *Uca deichmanni*, maintain this timing throughout the year as temperature, and thus the rate of embryonic development, vary. The mechanisms that allow such precision in the timing of the production of young are poorly known. A preliminary study suggested that when temperature decreases, *U. terpsichores* mate earlier in the tidal amplitude cycle such that larvae are released at the appropriate time. We tested this idea by studying the timing of courtship in *U. terpsichores* and *U. deichmanni* as temperature varied annually during two years, at 5 locations that differed in the temperature of the sediment where females incubate their eggs. *Uca terpsichores* courted earlier at locations where sediment temperature declined seasonally but not where sediment temperature remained elevated throughout the year. In contrast, clear shifts in courtship timing were not observed for *U. deichmanni* despite variation in sediment temperature. We discuss other mechanisms by which this species may maintain reproductive timing. These two species are likely to be affected differently by changes in the frequency and intensity of cold periods that are expected to accompany climate change.

## Introduction

Climate change is already affecting coastal marine environments. Among the myriad of effects are shifts in reproductive timing and an ensuing potential for alterations to community dynamics mediated by interspecific differences in responses and impacts [1], [2]. Most recent research on reproductive timing has been on the maintenance of, or changes in, the timing of the seasonal onset of reproduction in annually-breeding terrestrial organisms [3]–[5]. However, a large number of species, marine invertebrates in particular, reproduce multiple times per year. While many of these species do not breed when temperature is most variable, many continue to reproduce across large seasonal changes in temperature [6]–[8]. We know very little about how these organisms respond to seasonal or shorter-term temperature variation. Studies of species' responses to temperature variation can reveal the types of responses and temperature conditions that increase vulnerability to negative impacts on reproductive success and have the potential to alter community dynamics. Here we test whether two species of fiddler crabs change courtship timing in response to natural temperature variation.

Many species time reproduction relative to environmental cycles so that hatching occurs when conditions are most favorable for growth and survival of their offspring [9]–[11]. Reproduction is often linked to environmental cycles with periods that are unaffected by changes in temperature, such as lunar, tidal, diurnal or seasonal photoperiod cycles. However, variation in temperature can affect the ability of ectotherms to time the production of young relative to these cycles by affecting the rate their embryos develop [12], [13]. If such animals do not compensate for this effect of temperature on development, they risk producing young at times when their fitness would be reduced [9], [11], [14]–[19]. Hence, selection should favor flexible responses by adults that minimize potential timing errors caused by temperature variation.

Behavioral plasticity is one of the most effective ways in which organisms respond adaptively to variable environmental conditions [20]. Reptiles, insects and spiders mitigate the negative effects of variable conditions by being flexible in their activity, selection of habitat, and care of young [21]–[23]. Many intertidal crab species release their planktonic larvae approximately every two weeks during the largest amplitude nocturnal tides, when larvae can best avoid fishes that feed near the shore during the day [8], [24]. Evidence that this timing is important includes plasticity in the timing and intensity of reproduction in response to seasonal and spatial variation in tidal patterns, as well as changes in mate preferences and the timing of ovulation relative to the timing of mating [8], [25]–[28]. Several temperate species of fiddler crabs (genus *Uca*) have been shown to largely maintain their timing of release of larvae on large amplitude nocturnal ebb tides in spite of variation in temperature during the breeding season [29]. More recently, Kerr *et al*. [7] found that the tropical fiddler crabs *Uca terpsichores* and *U. deichmanni*, which breed year-round, sometimes make timing errors during the annual cold period that accompanies the upwelling of deep ocean water along the coast of the Eastern Pacific Ocean. However, even then, their timing of release of larvae is more accurate and synchronous than expected based on the effect of temperature on the rate that their embryos develop.

There are at least four, non-exclusive, behavioral mechanisms by which fiddler crabs minimize changes in the timing of larval release caused by temperature-induced changes in incubation period. Christy [30] presented preliminary evidence that *Uca terpsichores* courts several days earlier in the tidal amplitude cycle when the sea temperature declines during the annual period of cold water upwelling on the coast of Panama (January to April) [31]. Females of this species ovulate and begin incubation about 1.5 days after they court and mate, and they remain underground at a relatively fixed position until their eggs hatch two to three weeks later [32]. Plasticity in the timing of courtship, and the onset of incubation, can compensate effectively for temperature-induced changes in incubation period only if the sediment temperature where females incubate their eggs varies little during development. Indeed, the largest errors in timing of larval release by this species occurred when temperature changed after females had begun incubation [7]. Second, females of some species of *Uca* may delay mating or ovulation after they choose a mate and thereby delay the onset of incubation by a few days [30], [33], [34]. Such a delay in the start of embryonic development could permit females to adjust to increases but not decreases in temperature. Third, females may change the duration of incubation by choosing an appropriate burrow in which to mate and incubate their eggs [27], [35]–[37]. Fourth, Christy [30] speculated that females may behaviorally regulate the temperature at which their eggs develop throughout incubation by moving to appropriate microhabitats such as moving vertically along the thermal gradient within their burrow.

The primary objective of the present study was to verify plasticity in the timing of courtship in *Uca terpsichores* as temperature varies and to examine this possibility for the first time in *U. deichmanni*. We quantified male courtship behavior of both species across annual variation in sea and sediment temperature during two years and at several sites that have different patterns of variation in the temperature of the sediment. We also determined whether sediment temperature remains relatively stable for periods about equal to the duration of embryonic development during the season when sea temperature fluctuates rapidly.

## Materials and Methods

### Biology of study organisms

Our study species are often found on the same sandy beaches, with *Uca terpsichores* in the upper and *U. deichmanni* in the mid intertidal zone. Males of both species use claw-waving displays, rhythmic movements of their single large claw, to attract receptive females to their burrows to mate (Fig. S1, Video S1). The intensity of male courtship (number of males courting/area) is synchronized with female receptivity, ovary development and the timing of mating [38]–[40]. For *U. terpsichores*, male courtship intensity is also known to be synchronized with the timing of ovulation and onset of incubation [32], [41]. After the female ovulates in the male's burrow, the male leaves the burrow and the female incubates her eggs in a chamber ∼20 cm below the sediment surface. The duration of incubation for the two species depends on temperature. During the annual cold period in 2009, the incubation period ranged from 9–16 days for *U. deichmanni* and 14–21 days for *U. terpsichores* [7]. Peaks in courtship and hatching of larvae by both species occur approximately every two weeks mainly on the days with the larger amplitude tides [7], [30], [32], [39], [40], [42].

### Study site

**Figure 1:**
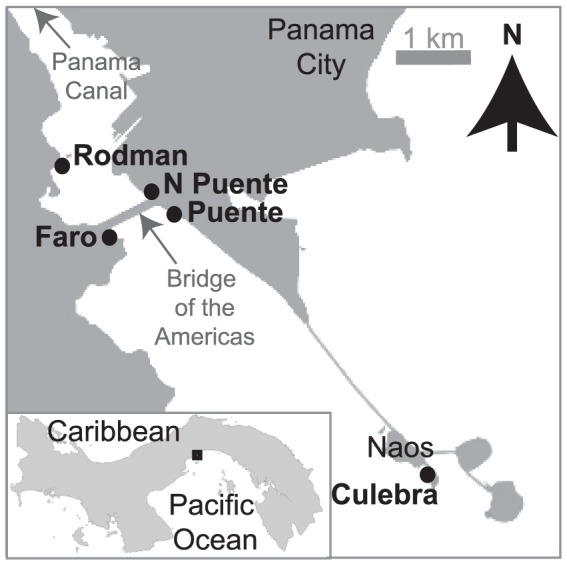
Study area. Location of study sites near the Pacific entrance to the Panama Canal in the Bay of Panama. Land is represented by gray shading and water is shown in white.

Between January 2008 and July 2009 we collected data on fiddler crab courtship and sediment temperature on five beaches within 5 km of one another at the Pacific entrance to the Panama Canal (Fig. 1). Research permits and access to study beaches were granted by the Autoridad de Recursos Acuáticos de Panamá (ARAP); Unidad Administrativa de Bienes Revertidos del Ministerio de Economía y Finanzas, el Servicio Nacional Aeronaval (access to the old Rodman Naval Base) and the Autoridad del Canal de Panamá (ACP). The tides in this area are semidiurnal and the largest amplitude tides occur on days when the tide is high just before sunrise and sunset. The tidal amplitude cycle has a period of 12 to 17 days and amplitudes range from ∼3 to 6 m (http://www.pancanal.com/eng/op/tide-tables.html). From May to December, the water temperature averages ∼29°C. Between January and April, wind-driven upwelling of deep ocean water causes a seasonal decrease in surface water temperature to ∼20 to 25°C [31]. The temperature of the sediment is influenced by the temperature of the sea, but differs among the five beaches and the habitats of the two species. The causes of these differences between beaches are beyond the scope of this study, but they are likely due to differences in beach aspect, slope, insolation, drainage, wind exposure, sediment composition and height of the habitat within the intertidal zone.

### Temperature data collection & analysis

Water and sediment temperature were recorded every hour throughout the study by *iButton* dataloggers (Maxim Embedded Datasystems). We sealed each *iButton* in three layers of polyethylene plastic using an Impulse MP-12 heat sealer. Water temperature was recorded at 1 m depth in the center of Culebra Bay (8°54′45″ N, 79°31′48″ W) (Fig. 1). Sediment temperature at 20 cm, the average burrow depth of ovigerous females, was recorded at each beach in the habitat of each species. Average daily temperature was used in all temperature analyses. Linear relationships between water temperature and sediment temperature were used to fill missing values in the sediment temperature time series. Missing values for the water temperature time series were estimated using the strong correlation between water and sediment temperature in *U. deichmanni* habitat at Culebra (R^2^ = 0.94 for 353 days of data for both time series).

The Panama Canal Authority defines upwelling conditions based on several indices, including when water temperature is less than 26.6°C. We adopt this temperature threshold as our definition of upwelling conditions. During this study the upwelling periods were Jan 26–May 12, 2008 and Jan 24–May 5, 2009. In April of 2008 and 2009 the water temperature exceeded 26.6°C for 21 and 6 days respectively, but then declined again before increasing and remaining at ∼29°C.

**Figure 2:**
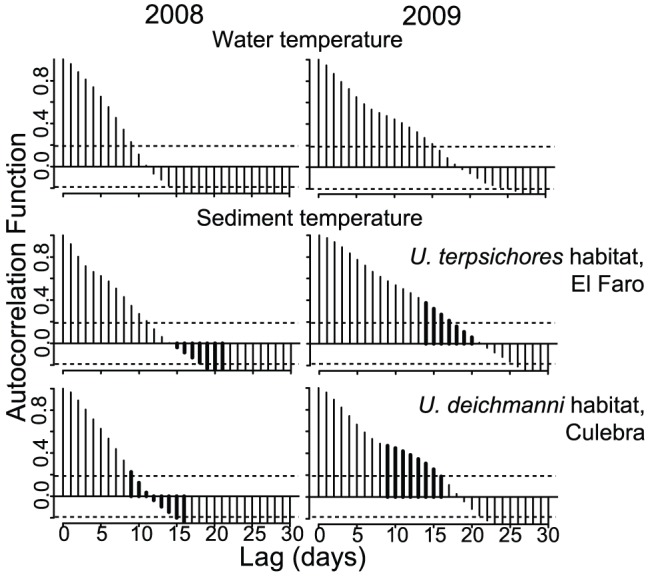
Temperature correlograms. Predictability in temperature during the upwelling season in both years expressed as the strength of the autocorrelation between the temperature on a given day and 1–30 days (lag) in the future. Water temperature (top row) was measured at 1 m depth in Culebra Bay. Sediment temperature was recorded at 20 cm depth in the habitat of each species at the coldest site for which we have data for both years (middle and bottom rows). Autocorrelation function values greater than the dashed horizontal line differ significantly from zero. Lags equivalent to the range of field measurements of incubation periods of both species during upwelling in 2009 are highlighted in bold (*Uca deichmanni*: 9–16 days, *U. terpsichores*: 14–21 days, Kerr et al. 2012, note that incubation period data is not available for 2008, but would have been shorter on average based on the relationship between temperature and incubation period). Significant autocorrelation at lags equivalent to the incubation period indicates that water temperature is predictable at the scale of the period of incubation.

Predictability of sediment temperature at the scale of an average incubation period of our species during the cold water upwelling season (*Uca deichmanni*  = 13.4 days ±1.72 SD and *U. terpsichores*  = 18.3 days ±3.09 SD, [7]) was assessed using autocorrelation. Correlograms of sediment temperature were produced in R Version 2.14.1 [43]. Ninety-five percent confidence intervals plotted on the correlograms were used to test the null hypothesis that the correlation for a given lag was not significantly different from zero. For each correlogram, we determined the maximum lag in days for which the time series maintained significant autocorrelation (ACF values greater than the 95% confidence interval threshold) (Fig. 2). We used this time period as an estimate of the time scale at which temperature is predictable. The sites with the largest variation in sediment temperature are those where crabs will experience the largest changes in incubation period and thus, will most need to make adjustments in courtship timing to avoid errors in timing of larval release. If temperature is predictable at these sites, changes in courtship timing could buffer changes in incubation period. However, low predictability in temperature at these sites will be more likely to cause errors in timing than at sites where the range in temperature change is small. Thus, correlograms from only the coldest sites of each species' habitat for which we have data during both years are discussed.

### Behavioral sampling

Counts of the number of courting males (waving their claw and not feeding, Video S1) in strip transects were conducted during diurnal low tides. Transects measured 1 m by 5 m in 2008. The density of *Uca deichmanni* was low near the end of the sampling period in 2008, so we doubled the transect size at all sites for both species to 2 m by 5 m in 2009. Transects were set out daily and crabs were observed through binoculars from approximately 3–5 meters away. Three counts were made at 3-minute intervals by visually scanning across the transect and rapidly assessing the behavior of each individual. The number of courting males was recorded and the average number of courting males was calculated for the day. Counts were made daily at all sites, except for 1–2 days of each tidal amplitude cycle during the neap tides when the low tides occurred at about dawn and dusk. The high tides on these days were often too low to inundate the habitat of *Uca terpsichores*. Very few crabs of either species were active on the surface during low tide on these days [39,40, K. Kerr pers. obs.]. Sampling was conducted at Culebra, Puente and El Faro (Fig. 1) between January 31 and July 25, 2008 and between January 22 and May 19, 2009 (3 sites/day). In 2009 we also sampled at North Puente and Rodman between January 22 and June 17 (5 sites/day). This schedule resulted in counts of courting males for 3 to 8 biweekly courtship cycles during each season at each site. We counted courting males between the time of the low tide and ∼30 minutes before the tide covered the habitat, a period during which the intensity of courtship by *U. terpsichores* is nearly constant [32]. The order in which we made the counts on the different beaches was randomized for each tidal amplitude cycle. Behavioral observations were made by several researchers over the course of the study. The consistency and accuracy of counts among observers was confirmed throughout the study by the close similarity of simultaneous counts between observers.

### Courtship timing relative to the tidal amplitude cycle

We used wavelet coherence analysis to examine the coherence in the fluctuations of the courtship and tidal amplitude time series. Wavelet analysis decomposes a time series into time and frequency space simultaneously; thus highlighting periodicity in the time series at time periods ranging from double the sampling frequency (2×1 day for our dataset) to one half the length of the total time series [44]. Wavelet coherence analysis extends wavelet analysis to provide the strength of correlation between two time series at multiple time scales across the length of the time series [44]–[46]. Wavelet analysis has important advantages for analyzing ecological time series compared to other methods: it does not assume that the time series is stationary (has a constant period length), and it reveals **when** changes in periodicity, or, in the case of wavelet coherence analysis, changes in the correlation between two time series, occurred [45], [46]. By quantifying the strength of correlation between the two time series at multiple time scales across the length of the time series, this analysis allowed us to determine if the periodicity and timing of courtship relative to the amplitude of the tide varied over time and among sites for each species [44]–[46].

Further, wavelet analyses are robust to different methods of filling missing data. Our time series consist of nearly six months of daily sampling at multiple sites during two years, making missing data as a result of bad weather (courtship ceases during heavy rain) or logistical problems inevitable. We estimated missing data using the average number of courting males for the 2 days after and one day before the missing counts. We then smoothed the time series by calculating a moving 3-day average and we stabilized the variance using a square root transformation. For the tidal amplitude time series, we calculated the daily nocturnal tidal amplitude of the previous night's ebbing tide using published tide tables (http://www.pancanal.com/eng/op/tide-tables.html). To match courtship intensity of a given cycle with the tidal amplitude of the next cycle (about 15 days later) when females release their larvae, we shifted the tidal amplitude time series one cycle earlier in time. We performed the analyses using Grinsted et al.'s wavelet coherence function in MATLAB (code is also available for R) [47], [48]. Statistical significance testing was conducted using Monte Carlo methods [47]. 1000 sets of surrogate time series with the same first order autoregressive (AR1, red noise) coefficients as our input time series were created. Correlations were calculated for each pair of surrogate time series and the significance level was estimated for each time scale [46], [47].

**Figure 3:**
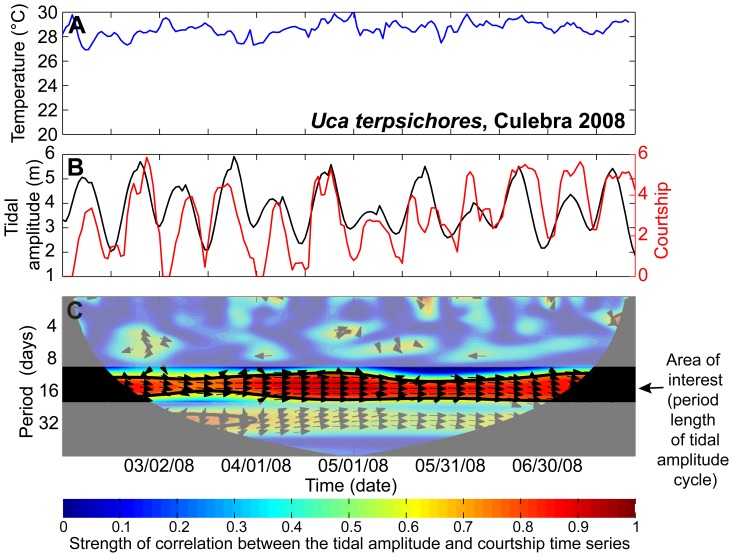
Wavelet coherence of courtship intensity and tidal amplitude. Time series of temperature (A), courtship intensity (square root of average number of males courting during the three scan samples, red line) and tidal amplitude (shifted one cycle earlier in time, black line) (B), and the wavelet coherence plot (C) for *Uca terpsichores* at Culebra for February to August 2008. In C, warmer colors represent stronger correlations between the courtship and tidal amplitude time series with dark blue indicating no correlation (0) and dark red indicating perfect correlation (1). When the correlation reaches a threshold level (0.5), arrows represent the phase relationship between the time series: right  =  in phase, left  =  anti-phase (180 degree offset between the time series), up or down  =  one time series is offset by 90 degrees from the other. The bold black line surrounding dark red areas indicates significant correlations between the two time series at the 5% level against red (AR1) noise. Data near the beginning and end of the time series can produce unreliable results and are thus covered by black on the plot (referred to as the “cone of influence” [47]). The time period of interest, one tidal amplitude cycle (12–17 days), is highlighted in bright colors. In subsequent figures, the wavelet coherence plots exclude periods outside this area of interest.

Results of the wavelet coherence analysis are presented in wavelet coherence plots (Fig 3C, *Uca terpsichores* at Culebra in 2008) showing the strength and statistical significance of the correlation between the courtship and tidal amplitude time series, as well as the phase relationship between the time series. Data near the beginning and end of the time series can produce unreliable results and are thus covered by the black “cone of influence” on the plot [47]. Since we are interested in whether courtship timing varies with respect to the tidal amplitude cycle across seasonal changes in temperature, we focus on the period of one tidal amplitude cycle (12–17 days, shown in bright colors in Fig. 3C). Variation in the strength of the coherence between the two time series at this time period indicates a shift in timing or breakdown in phase-locking, or synchrony, of the two time series. Thus, in Fig. 3C the dark red band surrounded by the black line between 14 to 17 day periods (y-axis) spanning the length of the sampling dates (x-axis) indicates a significant correlation between courtship and tidal amplitude throughout the time series for *U. terpsichores* at Culebra in 2008. Arrows directed towards the right indicate that the time series are phase-locked and in phase. Period lengths outside the range of the tidal amplitude cycle have been cut from all other wavelet coherence plots.

### Median courtship timing relative to sediment temperature

To examine the relationship between courtship timing and sediment temperature across all sites, we used a Generalized Linear Model (gamma error distribution, log link) to determine the effect of sediment temperature, species, and their interaction on the number of days between the median day of courtship and the target date for larval release (largest amplitude nocturnal tide of the following tidal amplitude cycle). Median courtship day was defined as the day on which the cumulative proportion of the total number of courting males observed during a courtship cycle reached 0.5. Start and end dates of courtship cycles were defined by minimum courtship values. Average sediment temperature at 20 cm depth during the preceding tidal amplitude cycle (minimum amplitude tide to minimum amplitude tide) was used for this analysis since this is when crabs assess when to begin courtship. The analysis was conducted in R Statistical Computing Environment (R version 2.14.1).

## Results

### Variation in temperature

The annual seasonal decline in sea temperature and the duration of the decline were greater in 2009 than in 2008 (Fig. S2). The temperature of the water recorded in these years was typical of the annual long-term variation in temperature for the Gulf of Panama [STRI Environmental Monitoring Program, Pacheca dataset, 31]. In general, the temperature of the sediment in the lower, wetter habitat of *Uca deichmanni* followed the temperature of the water more closely than did the temperature of the sediment in the higher, drier habitat of *U. terpsichores* (Fig. S2). However, sediment temperature differed among sites in the habitats of both species (Fig. S2). For example, the temperature of the sediment in both species' habitats at Rodman and El Faro was relatively low and tracked variation in water temperature closely (Fig. S2). In contrast, at Culebra the sediment in *U. terpsichores* habitat was often the warmest of all sites while the sediment in *U. deichmanni* habitat was the coolest (Fig. S2). These differences in sediment temperature among sites for each habitat allowed us to examine local effects of temperature on reproductive timing independent from the possible effect of time of season.

### Correlations between sediment temperature at the time of mating and during incubation

A close correlation in the temperature of the sediment during the period when crabs determine when to court, and during the period of incubation of the eggs, is required if crabs are to appropriately time release of larvae by changing when they mate. Increases in the duration of incubation and magnitude of potential errors in timing would be greatest at sites that get the coldest. Thus, we focused on the coldest sites for which we have data from both years, Culebra for *Uca deichmanni* and El Faro for *U. terpsichores*, when examining whether sediment temperature before courtship commences is a reliable cue for sediment temperature during incubation, Temperature was significantly autocorrelated in *U. deichmanni* habitat at Culebra for 9 days during the warmer, but more variable 2008 upwelling season, and 15 days in 2009 (Fig. 2). Significant autocorrelation in temperature extended 1 to 2 days longer in *U. terpsichores* habitat at El Faro (Fig. 2). Thus, during colder upwelling seasons, such as in 2009, when adjustment to lower temperature is more important, sediment temperature on a given day is a reliable cue for the temperature of the sediment for the duration of incubation for *U. deichmanni* (∼13 days on average during upwelling), and for most of the duration of incubation for *U. terpsichores* (∼18 days on average during upwelling) (Fig. 2).

### Courtship relative to the tidal amplitude cycle

**Figure 4:**
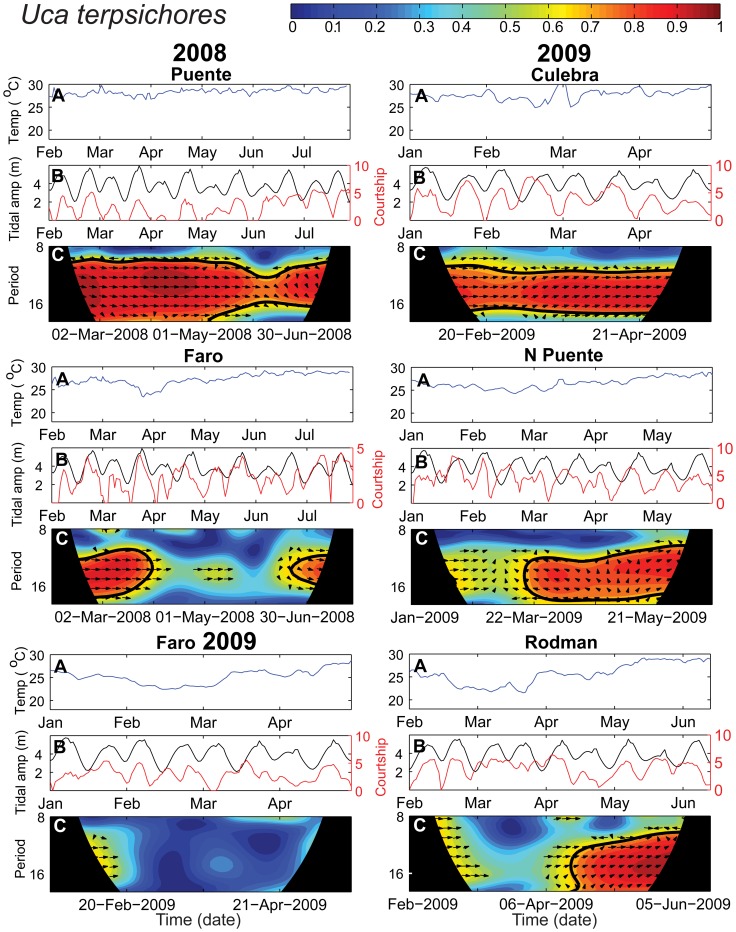
Temperature, tidal amplitude and *Uca terpsichores* courtship intensity. Wavelet coherence results for *U. terpsichores* for two sites in 2008 and four sites in 2009. See the Figure 3 caption for an explanation of the panels A, B and C. Only the period lengths (y-axis) of approximately one tidal amplitude cycle (12–17 days), the area of interest, and a buffer of a few days on either side are shown in plot C.

**Figure 5:**
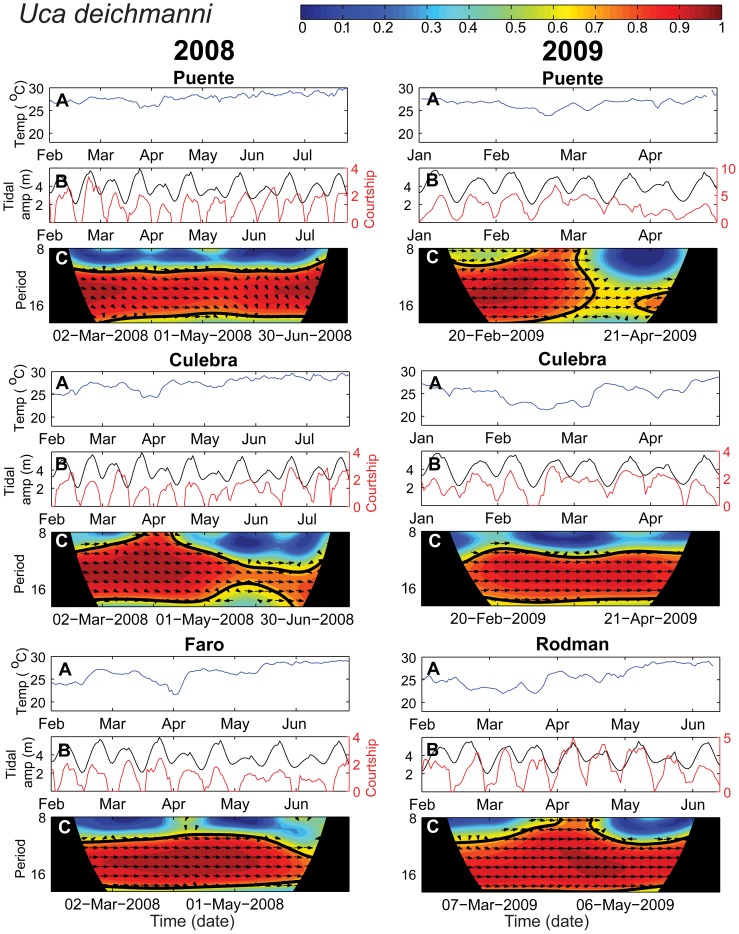
Temperature, tidal amplitude and *Uca deichmanni* courtship intensity. Wavelet coherence results for *U. deichmanni* courtship and tidal amplitude for three sites in 2008 and 2009. Panels A, B and C as explained for Figure 3. Only the period lengths (y-axis) of approximately one tidal amplitude cycle (12–17 days), the area of interest, and a buffer of a few days on either side are shown in plot C.

As expected, courtship intensity varied strongly with tidal amplitude for both species (Figs 3–5 B). For *Uca terpsichores*, peaks in courtship generally coincided with peaks in tidal amplitude during the warm season, and throughout sampling at Culebra and Puente, where the sediment temperature remained high even when the water temperature decreased (Figs 3 & 4, A & B). This strong in-phase relationship between courtship and tidal amplitude is evident in the wavelet coherence plots as a significant correlation between the time series (a red band surrounded by a black line) between periods of 12 to 17 days (y-axis) that spans the length of the sampling period (x-axis) at Culebra and Puente in both years (Figs 3 & 4, C, Puente 2009 data not shown). However, there is variation in this relationship both temporally and spatially for this species. At El Faro and Rodman, the correlation between courtship and tidal amplitude became weak and non-significant beginning when sediment temperature decreased in late March in 2008 and February in 2009, but was significant during non-upwelling conditions (Fig. 4A & C). At Rodman, where *U. terpsichores* habitat got the coldest during the strongest period of upwelling, this species failed to exhibit semilunar cycles of courtship between late-February and mid-April (Fig. 4A & B). At El Faro, the coherence between the time series differs between 2008 and 2009 in concordance with the difference in temperature between the years. In 2008 it was relatively warm during the upwelling period and we found a significant correlation between the two time series for part of the season. In 2009, when the temperature declined, the time series were not significantly correlated. At North Puente, temperature declined smoothly and the correlation between the time series was always greater than 0.5, but the phase relationship between the two time series shifted when the temperatures were lower between January and May. We conclude that *U. terpsichores* males altered courtship timing for some periods of the cold-water season at sites where sediment temperature decreased.

For *Uca deichmanni*, peaks in courtship intensity coincided with peaks in tidal amplitude at all sites throughout the study despite considerable variation in temperature within and between sites (Fig. 5 A&B). Courtship and tidal amplitude were significantly correlated throughout the time series (across both seasons) at all sites as indicated by the consistent red band delimited by the black line across the wavelet plots (Fig. 5C). Hence, no change in timing of courtship was detected for this species despite variation in temperature in its habitat.

### Median courtship timing relative to sediment temperature

**Figure 6:**
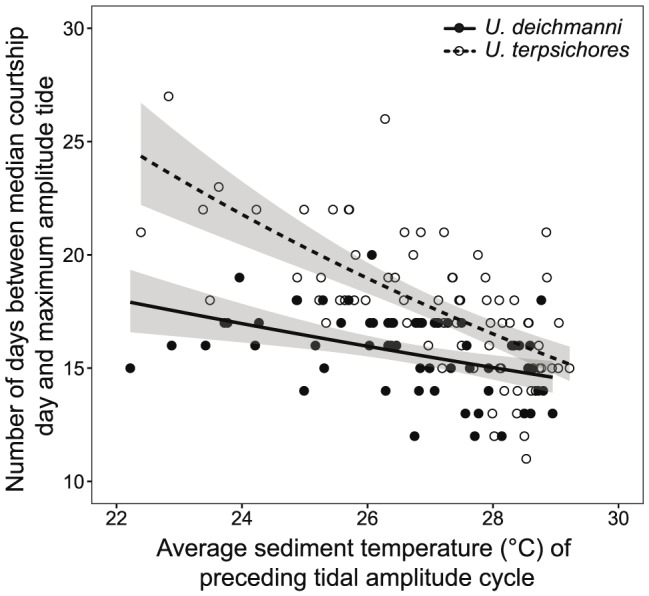
Median courtship timing vs sediment temperature. The number of days between the median courtship day and the night in the following tidal amplitude cycle with the largest amplitude tide (target day for larval release) versus sediment temperature for *Uca terpsichores* (N = 74) and *Uca deichmanni* (N = 59) (Gamma GLM, log link, Temperature: F = 53.17, P<<0.001, Species: F = 44.57, P<<0.001, Temperature * Species: F = 8.71, P = 0.004). Median courtship day was defined as the day when the cumulative proportion of all males observed courting during the courtship cycle surpassed 0.5. Sediment temperature is the average temperature at 20 cm depth during the preceding tidal cycle (minimum amplitude tide to minimum amplitude tide).

Median courtship timing relative to the maximum amplitude tide depended on sediment temperature for both species, with median courtship occurring earlier (larger number of days to the maximum amplitude tide) when temperature was lower (Fig. 6, GLM (gamma, log link), F_temp_ = 53.17, P_temp_<<0.001). Further, the relationship between courtship timing and temperature differed significantly between the species, with a larger change in courtship timing for a given change in temperature for *Uca terpsichores* (Fig. 6, GLM (gamma, log link), F_species_ = 44.57, P_species_<<0.001, F_species*temp_ = 8.71, P_species*temp_ = 0.004). The slight temporal shift in courtship by *U. deichmanni* was thus, significantly smaller than the shift observed for *U. terpsichores*, and was not resolved by our wavelet analysis.

## Discussion

Intertidal organisms that respond flexibly to environmental variation in space and time can maintain strong reproductive cycles and precisely time their release of larvae. Large differences in temperature and in some cases tidal patterns on local to regional scales mean that most intertidal organisms with a planktonic larval stage are unlikely to experience the same conditions as did their parents. Fiddler crab adults, and even transplanted embryos, show great flexibility in their ability to adjust to local tidal conditions and maintain timing of hatching [25], [49]–[52]. However, the mechanisms these crabs use to maintain timing in response to variation in temperature are largely unknown [30]. Here, we examined the possibility that plasticity in the timing of mating may allow fiddler crabs to maintain adaptive timing of release of larvae as temperature varies. Our focal species differed in their response in courtship timing to changing temperature.

For *Uca terpsichores*, the correlation between courtship intensity and tidal amplitude varied both spatially and temporally with variation in temperature. Further, a significant negative relationship between median courtship timing and temperature was observed. These results demonstrate that this species changes when it courts in response to changing temperature. Shifts in the timing of mating that account for the effect of temperature on development period have been documented for many organisms that breed seasonally. However, such shifts by organisms that reproduce multiple times per year have been described only for a few species of fish [53]–[55] and may be inferred for others [56], [57]. Given that many intertidal species reproduce synchronously and periodically across temporal and spatial variation in temperature, plasticity in the timing of mating as a mechanism to maintain the timing of hatching with respect to the tidal amplitude cycle may be more common than currently recognized.

Flexible responses to current cues are expected when cues are reliably correlated with future conditions [58]. As shown in other studies, we found that current temperature usually is a reliable cue for temperature during the incubation period [59]. We showed previously that *U. terpsichores* reduced errors in its timing of larval release relative to the errors expected based on the effect of temperature on incubation period [7]. Our results here indicate this species did so by changing when it mated in response to changing temperature. The scale of the shift of ∼1.3 days/°C is consistent with the change in incubation period Kerr *et al*. observed in the field (∼1.2 days/°C) [7]. Yet, changes in temperature during incubation did occur and produced errors in timing by *U. terpsichores* [7]. While this predictive strategy to maintain reproductive timing may be underreported, it is subject to error and may be restricted to species that are unable to adjust to temperature once incubation has begun.

Despite strong spatial and temporal variation in temperature in their habitat, we did not detect a change in the timing of courtship for *Uca deichmanni* with our wavelet coherence analysis. A small shift in timing was evident when all sites were pooled in the regression analysis. The scale of this shift (∼0.5 days/°C) is not sufficient to compensate for increases in incubation period observed in the field (∼0.8 days/°C) or the laboratory (∼1.0 days/°C) [7]. Yet, this species released larvae highly synchronously during the large amplitude tides despite variation in temperature of up to ∼6°C across seasons [7]. Thus, this species must use additional methods to adjust to temperature variation.

Females may maintain their reproductive timing as temperature varies by selecting the temperature at which their embryos develop. The size of male fiddler crabs, the diameter of their burrows, and burrow temperature are correlated. Females of some fiddler crab species that live in muddy intertidal habitats adjust their size-based preferences for mates, and thus burrow diameter and temperature, depending on the temperature or time remaining until the optimal period for larval release [27], [35]–[37]. This mechanism for adjusting incubation duration is unlikely to operate in the species we studied, which live in sandy sediments. In these habitats, the upper shaft of crabs' burrows collapse when the burrows are covered during high tide thus decoupling choice of mates from choice of incubation temperature as affected by burrow diameter.

Alternatively, females may behaviourally regulate incubation temperature. If, like *Uca terpsichores*, ovigerous females do not open their burrows after each high tide they will remain at a fixed depth in the sediment throughout incubation and thus be exposed only to the temperature of the sediment at that depth [30]. In *U. terpsichores*' upper intertidal, sandy, well-drained habitat, burrow shafts collapse upon immersion by the tide. If females opened their burrow during low tides they would likely expose their eggs to a risk of desiccation, thus preventing them from accessing microhabitat variations in temperature. *Uca deichmanni*, however, live in wetter sediment in the mid-intertidal zone. Ovigerous females occasionally open and remove sand from their burrows during low tide and then plug only the upper portion, leaving an open vertical chamber ≥10 cm long. Sediment temperature varies most near the surface, with maximum daily temperatures occurring after the daytime low tide when the surface of the sediment is heated by the sun (Fig. S3) [60]–[63]. We suggest that females may move vertically through the thermal gradient in the shaft they create, and in this way, control the temperatures to which their embryos are exposed. While there is evidence that marine organisms behaviorally regulate their own body temperature [64]–[68], regulation of the temperature at which their embryos develop is not well-documented. For fiddler crabs, this method of adjustment is most likely to be observed in species with stable burrow shafts, where a gradient can most easily be accessed, or in species like *U. deichmanni*, where burrows collapse but females re-open the burrow and re-establish the burrow shaft. This method of adjustment to environmental variation would be particularly advantageous when embryos are unpredictably exposed to conditions that affect their development or survival, even if they are uncommon [23].

Environments can vary so much that reproduction is suspended or suppressed when conditions become unfavorable for the growth and survival of offspring. Seasonal reproduction is the most common example of this. However, some crabs and fish demonstrate a more subtle response, altering the level or intensity of reproduction to environmental conditions [26], [28], [39], [40], [56]. *Uca terpsichores* males courted less when variable or low temperatures coincided with lower amplitude tides when larvae would be released (Figs 3 & 4). For crabs, modulation of reproductive activity from cycle to cycle results in the release of larvae when tides are greater in amplitude [26], [39], [40]. At our sites, modulation of courtship intensity among tidal amplitude cycles was most evident during the cold water upwelling season. Large daily fluctuations in temperature or low average temperatures combined with lower amplitude tides may prove too risky for females to invest in egg production and incubation.

Changes in temperature may also result in changes in synchrony of life history events: synchrony is expected to increase with temperature [69]. In the Bay of Panama, low synchrony in spawning by sergeant major damselfish *Abudefduf troschelii* was reported during cold water upwelling but the cause of this pattern was not determined [57]. On the coast of California, low synchrony in larval release by intertidal crabs occurs when the water temperature is low and variable [70]. Low synchrony in courtship timing by *Uca terpsichores* during the coldest conditions at Rodman, our coldest site, is consistent with these studies. As the magnitude of changes in the timing of mating that are required to maintain timing of hatching increases, variation among females in the timing of their sexual receptivity should also increase. Low synchrony among females in receptivity and hence in courtship, mating and the onset of incubation, likely contributed to the low synchrony of larval release we observed at this site for several cycles during upwelling [7].

## Conclusions

The effects of variable temperature on diverse aspects of the biology of ectothermic organisms are increasingly being recognized, especially for reptiles and amphibians [e.g. 71,72,73]. Many studies have examined changes in reproductive timing with changes in temperature, especially for seasonally breeding organisms, but few have focused on both spatial and temporal variation in reproductive timing of more than one species [74], [75]. Using this approach, we provide evidence of differing adjustments in reproductive timing in response to variable temperature by two tropical marine species in the field. Along some coasts that experience upwelling of cold water, climate change is expected to result in larger, more prolonged decreases in sea surface temperature, or more frequent changes in temperature [76]–[79]. The different responses to temperature variation demonstrated by these two species, and the resulting constraints on their ability to adjust, mean that they may be affected differently by climate change. Increased temperature variability, and thus decreased predictability, will likely result in more frequent errors in timing of release of larvae by *Uca terpsichores* than by *U. deichmanni*. If climate change causes more intense but prolonged coldwater upwelling events, *U. terpsichores* should be able to adjust, but even if *U. deichmanni* females regulate incubation temperature behaviorally, the limits of their thermoregulatory ability may be surpassed [7]. The effects of errors in reproductive timing by fiddler crabs on population and community dynamics are currently unknown, but the range of methods of adjustment that maintain reproductive timing in this group emphasize its importance.

## Supporting Information

Figure S1 **Fiddler crab reproductive cycle.** A. Males attract receptive females to their burrows by waving their single greatly enlarged claw. B. Mating occurs in the male's burrow. C. After mating and ovulation, the male leaves and the female incubates her eggs in the terminal chamber of the burrow for about two weeks. D. When her eggs are ready to hatch, the female ascends to the surface and releases her larvae near the time of high tide, usually at night, on the days with large amplitude tides. E. Larvae that hatch at night escape predation from abundant small fish that feed visually during the day. Illustration by J. Luque.
(TIF)

Figure S2 **Water and sediment temperature.** Daily averages of water temperature at 1 m below surface in Culebra Bay (upper panel) and sediment temperature at 20 cm depth at each site (all other panels) in *Uca terpsichores* (thin solid line) and *Uca deichmanni* habitat (dark dashed line). Temperatures were recorded hourly by *iButton* data loggers.
(PDF)

Figure S3 **Vertical temperature profile.** Daily minimum, maximum and average sediment temperatures in *Uca deichmanni* habitat at Culebra from 3 depths: 0–5 cm, 10–15 cm, 20 cm. Temperatures were recorded hourly by *iButton* data loggers.
(PDF)

Video S1 ***Uca terpsichores*** **courtship display.** A male *Uca terpsichores* courting (waving his enlarged cheliped) at El Faro, near the Bridge of the Americas, Pacific coast of Panama.
(MOV)
