# Supplementary figures and images for: Reproducing on Time When Temperature Varies: Shifts in the Timing of Courtship by Fiddler Crabs
Source: PLoS One. 2014 May 15;9(5):e97593. doi: 10.1371/journal.pone.0097593 (PMC4022618; doi:10.1371/journal.pone.0097593)

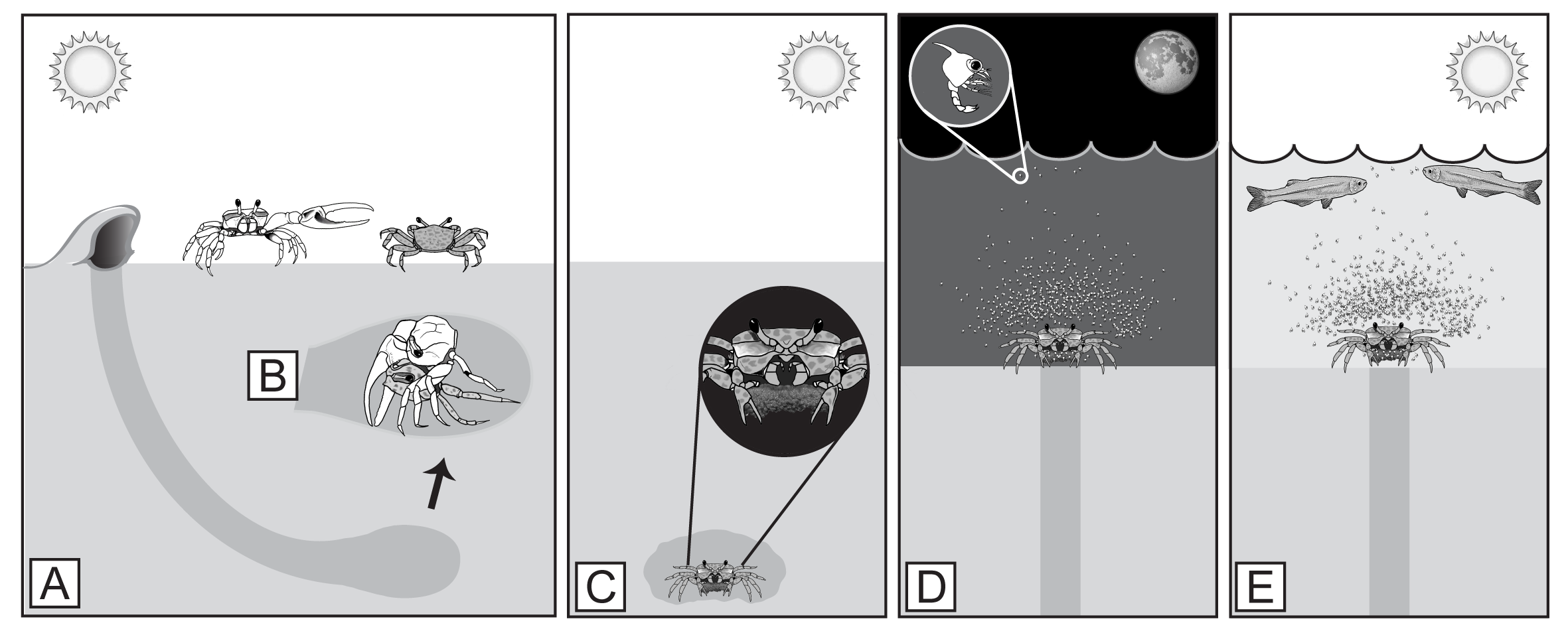

Supplement: Figure S1 — Fiddler crab reproductive cycle. A. Males attract receptive females to their burrows by waving their single greatly enlarged claw. B. Mating occurs in the male's burrow. C. After mating and ovulation, the male leaves and the female incubates her eggs in the terminal chamber of the burrow for about two weeks. D. When her eggs are ready to hatch, the female ascends to the surface and releases her larvae near the time of high tide, usually at night, on the days with large amplitude tides. E. Larvae that hatch at night escape predation from abundant small fish that feed visually during the day. Illustration by J. Luque. (TIF) [file pone.0097593.s001.tif]

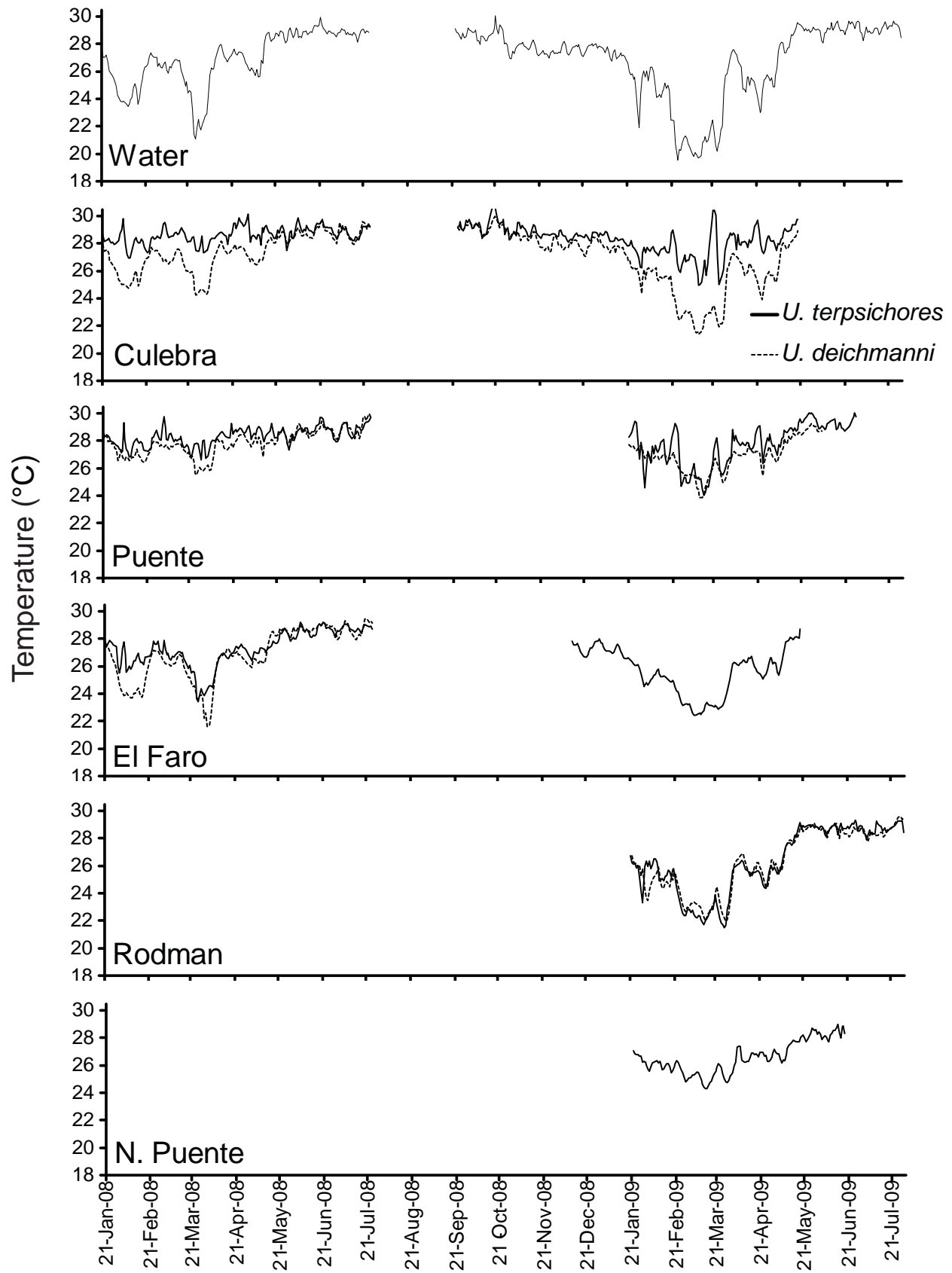

Supplement: Figure S2 — Water and sediment temperature. Daily averages of water temperature at 1 m below surface in Culebra Bay (upper panel) and sediment temperature at 20 cm depth at each site (all other panels) in Uca terpsichores (thin solid line) and Uca deichmanni habitat (dark dashed line). Temperatures were recorded hourly by iButton data loggers. (PDF) [file pone.0097593.s002.pdf]

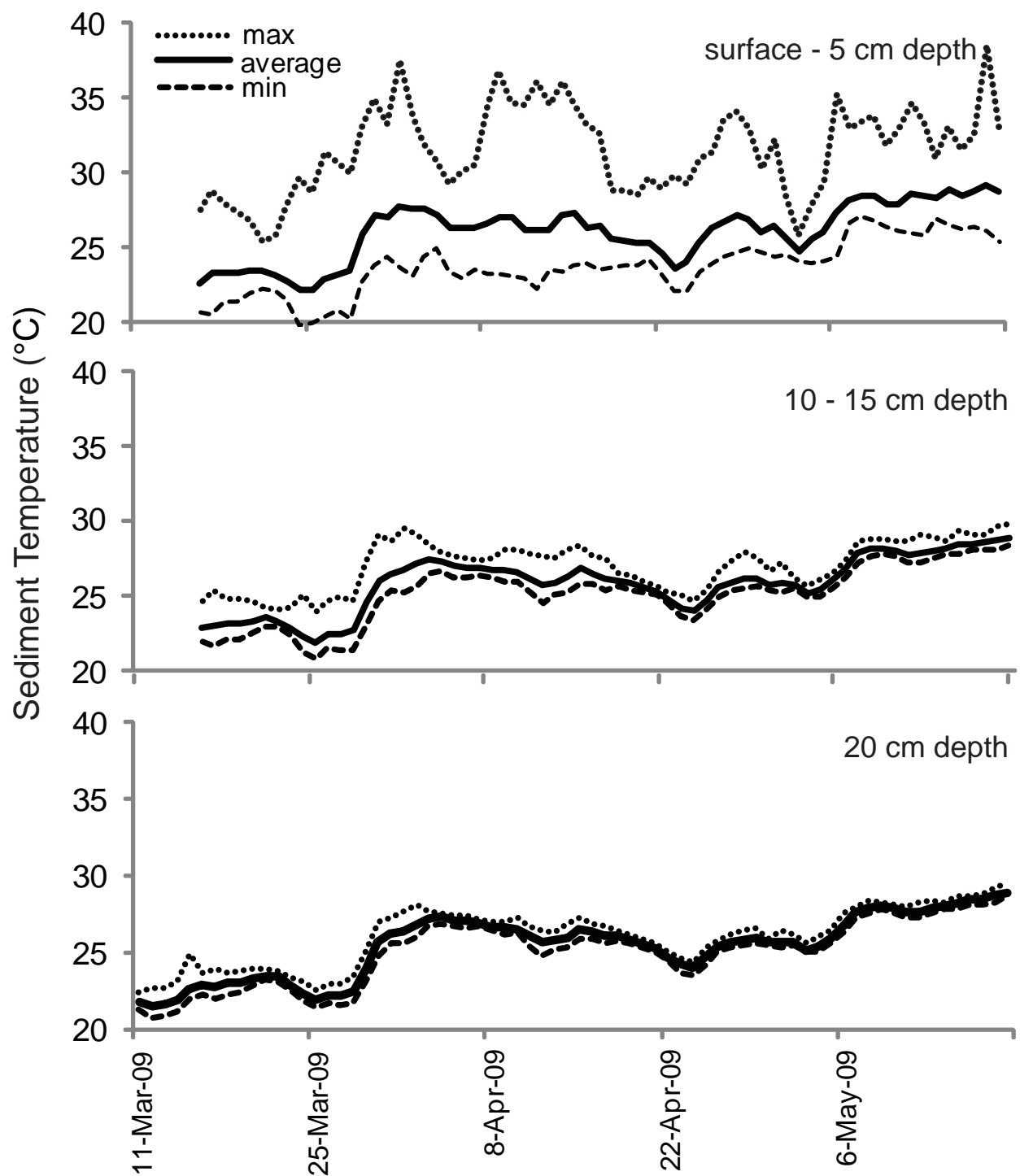

Supplement: Figure S3 — Vertical temperature profile. Daily minimum, maximum and average sediment temperatures in Uca deichmanni habitat at Culebra from 3 depths: 0–5 cm, 10–15 cm, 20 cm. Temperatures were recorded hourly by iButton data loggers. (PDF) [file pone.0097593.s003.pdf]
